# Biology and Treatment of High-Risk CLL: Significance of Complex Karyotype

**DOI:** 10.3389/fonc.2021.788761

**Published:** 2021-11-29

**Authors:** Thomas Chatzikonstantinou, Christos Demosthenous, Panagiotis Baliakas

**Affiliations:** ^1^ Hematology Department—Bone Marrow Transplantation (BMT) Unit, G. Papanicolaou Hospital, Thessaloniki, Greece; ^2^ Department of Immunology, Genetics and Pathology, Science for Life Laboratory, Uppsala University, Uppsala, Sweden; ^3^ Department of Clinical Genetics, Uppsala University Hospital, Uppsala, Sweden

**Keywords:** CLL (chronic lymphocytic leukemia), prognosis, prediction, high-risk, complex karyotype (CK), cytogenetic complexity

## Abstract

Several reports highlight the clinical significance of cytogenetic complexity, namely, complex karyotype (CK) identified though the performance of chromosome banding analysis (CBA) in chronic lymphocytic leukemia. Indeed, apart from a number of studies underscoring the prognostic and predictive value of CK in the chemo(immune)therapy era, mounting evidence suggests that CK could serve as an independent prognosticator and predictor even in patients treated with novel agents. In the present review, we provide an overview of the current knowledge regarding the clinical impact of CK in CLL, touching upon open issues related to the incorporation of CK in the clinical setting.

## Introduction

Chronic lymphocytic leukemia (CLL) is the most common hematological malignancy among the elderly in the western world, characterized by clonal growth of mature, CD5+ B lymphocytes in the bone marrow, peripheral blood, and secondary lymphoid organs (1). The clinical course of CLL is extremely variable, ranging from asymptomatic to highly aggressive, which likely reflects the underlying biological heterogeneity (2). A great number of clonal- and patient-related features with prognostic and/or predictive value have been identified over the last decades, in an effort to optimize the management of CLL (3–9).

Aberrations detected with Fluorescence *in situ* hybridization (FISH), namely the Döhner hierarchical model, have for the last 20 years served as the backbone of CLL diagnostics, dictating the treatment choice, together with the mutation status of *TP53* gene and also the segregation into mutated- and unmutated-CLL (M-CLL and U-CLL respectively) (10, 11). Nevertheless, FISH analysis cannot provide a comprehensive overview of the genomic background of the clone, something that can be accomplished with other methodologies, such as chromosome-banding analysis (CBA), chromosome microarray analysis (CMA) or genome-wide analysis (12–14). A major advantage of these methods, especially CBA, is the identification of complex karyotype (CK) which according to recent reports is a significant prognostic feature with the potential of becoming also a novel predictive biomarker (4, 15). A number of methodological issues, mainly the obtainment of adequate number of representative metaphases, have been overcome with the application of specific culture protocols, making CBA a robust and reproducible method (16–18).

In CLL similarly to other hematological malignancies, CK is defined by the presence of ≥3 numerical or structural abnormalities in ≥2 metaphases in the same clone. CK is reported in 10–20% of untreated patients with CLL and in 8% of patients with monoclonal B lymphocytosis (MBL) (4, 15, 18, 19). The association of CK with advanced-stage disease, U-CLL, *TP53* mutations, adverse FISH abnormalities (del(17p) and/or del(11q)), and telomere dysfunction (15, 20), suggests a potential role of chromosomal aberrations early in the disease development. CK development has been proposed to arise in a 4-phase process in U-CLL: enhanced response of lymphocytes to antigens leading to stimulation of intracellular B-cell-receptor (BCR) signaling and proliferation; telomere shortening during each cell division to a critical point; inactivation of genes implicated in DNA repair (e.g., TP53, ATM), ubiquitin-mediated degradation of oncoproteins (e.g., FBXW7), and to the inflammatory pathway (e.g., MYD88) and; increased genomic instability and risk of chromosome break events (21).

In the present review, we focus on the prognostic and the predictive value of CK in CLL, while we further discuss future perspectives regarding the potential role of CK in the management of CLL as well as issues related to the applied methodology and interpretation of eventual findings.

## Prognostic Significance of CK in CLL

The prognostic value of CK in CLL has been mainly assessed in retrospective studies of both untreated and treated patients, mostly in the era of chemo(immune)therapy. Juliusson et al. first reported that patients with indolent lymphomas (including CLL) and three or more cytogenetic aberrations exhibited dismal clinical outcome compared to patients with normal karyotype (22). In a later CLL-specific cohort, it was reported that three or more aberrations, were linked to shorter overall survival (OS), while specific structural abnormalities, such as aberrations involving 14q32 and trisomy 12 correlated with worse prognosis (23, 24). The advent of specific culture protocols with the addition of mitogens, namely CD40 ligand (CD40L) or CpG-oligonucleotide DSP30 plus interleukin-2 (IL-2) allowed the performance of large cytogenetic studies that further highlighted the association between CK and inferior clinical outcome in CLL. Mayr et al. reported that patients with CLL and CK had a shorter treatment free survival (TFS) and OS compared with patients with fewer aberrations, especially if accompanied by the presence of translocations (25). In a seminal study including 506 patients with CLL, CK was detected in 16% of the cohort, while interestingly paired analysis with FISH indicated that the two methods complement each other and that their combination provides a far more comprehensive genetic characterization than each assay alone (26).

Despite not being part of the standard diagnostic algorithm in CLL, several institutions included CBA in the CLL work up. This approach allowed the performance of the first large scale retrospective study with the inclusion of 1,001 patients with CLL, where CK was reported in 16% of the cohort at the time of CLL diagnosis (15). Furthermore, CK was, as in previous studies, associated with U-CLL, high expression of CD38, del(11q) and del(17p), while it was also an independent prognosticator for shorter time to first treatment (TTFT) and OS. In addition, it was suggested that the presence of ≥5 aberrations correlated with an even worse clinical outcome, however, the number of the patients in that subgroup was relatively low precluding from definite conclusions. Interestingly, the impact of CK was not affected by the presence of translocations, neither balanced nor unbalanced.

In a cohort with fewer patients, Rigolin et al. reported that the co-existence of CK and unbalanced rearrangements was associated with inferior TTFT and OS. In that study though, cases with CK and unbalanced rearrangements were enriched for TP53 aberrations (deletion and/or mutation). That said the negative impact of unbalanced rearrangements was independent of TP53 status (27). The association of TP53 status and CK was further evaluated by Puiggros et al. (28), who within a cohort of 1,045 patients with CLL detected 99 (10%) with CK. Once more, CK was associated with del(17p) and del(11q) [del(17p) and del(11q) defined as high-risk (HR) FISH], as well as with shorter TTFT and OS. Patients with CK and HR FISH exhibited the worst overall prognosis. However, the clinical outcome of patients with CK was not affected by the presence of HR-FISH, suggesting that other clinicobiological features than TP53 status may also contribute to the poor prognosis of CLL with CK.

Further insight on the prognostic value of CK in CLL and the impact of TP53 aberrations within this group was obtained in a large multi-institutional retrospective study, performed by the European Research Initiative on CLL (ERIC), which included more than 5,000 patients (4). The major conclusion of this study was that not all CKs in CLL are equivalent. CK segregated in three subgroups: low-CK (three aberrations), intermediate-CK (four aberrations) and high-CK (≥5 aberrations). Only patients with high-CK exhibited uniformly dismal clinical outcome irrespectively of other features including TP53 status. In contrast, low-CK and intermediate-CK was associated with unfavorable prognosis only in the presence of TP53 aberrations. Interestingly, 20% of patients with high-CK were lacking TP53 aberrations even when evaluated with sensitive high-throughput sequencing. Furthermore, among patients with CK, those carrying +12,+19 exhibited indolent clinical courses, confirming previous reports which suggested that CLL with +12,+19 represents a distinct subset with unique clinicobiological features (29, 30). In conclusion, the largest study thus far on CK in CLL highlights once more the heterogeneity of CLL even within this subgroup of patients, which for decades was considered as homogeneous both biologically and clinically.

## Predictive Significance CK in CLL

It is strongly suggested that CK is an unfavorable predictive marker among patients with CLL treated with chemo(immune)therapy (31–33). It should be however noted that this issue has not been addressed properly, since for many years CBA was not included in the standard work-up of clinical trials in CLL. Thus, it is still unclear if CK is indeed an independent predictor for patients treated with chemo(immune)therapy or the reported effect of CK is the result of a joint impact of CK and other unfavorable biomarkers, namely TP53 aberrations and U-CLL.

The predictive value of CK in CLL becomes even more relevant as the treatment of CLL is shifting drastically towards novel agents, i.e., B cell signaling kinase inhibitors and the Bcl-2 inhibitor venetoclax. Several studies have attempted to address this issue in the context of clinical trials and assess the potential utility of CBA as a predictive factor. Interestingly, cytogenetic complexity has been even assessed with the advent of CMA. In the great majority of these studies, CK was considered as one homogeneous group, defined by the presence of ≥3 cytogenetic abnormalities without taking into account the differentiation to low-CK, intermediate-CK and high-CK (**Table 1**).

**Table 1:** Randomized control trials that assessed the impact of complex karyotype in chronic lymphocytic leukemia.

Study	Number of patients	Type of treatment	Line of treatment	Definition of CK	PFS/OS	Comment
Brown et al. (34)	Total: 195 CK: 39	Ibrutinib	≥ 2nd line	≥ 3 cytogenetic abnormalities	**18-mo PFS**: 72%	PFS with Ibrutinib was similar regardless of the presence of CK.
	Total: 196 CK: 33	Ofatumumab			**18-mo PFS:** 0%	
Woyach et al. (35)	Total:333 CK: 99	Ibrutinib ± Rituximab	1^st^ line	≥ 3 cytogenetic abnormalities	NA	CK did not influence Ibrutinib-induced PFS in 1st line, HR = 1.01 (95% CI 0.68-1.51, p = 0.95)
	Total: 166 CK: 44	Bendamustine plus Rituximab				
Kreuzer et al. (36)	Total: 127 CK: 50	Idelalisib plus Rituximab	≥ 2nd line	≥ 3 cytogenetic abnormalities	**Median OS:** 28.3 [16.6, NR] months **Median PFS**:20.9 (8.5, NR) months	In the Idelalisib plus Rituximab arm, no significant difference in OS was noted between patients with or without CK.
		Rituximab plus placebo			**Median OS:** 9.2 [16.6, NR] months	
Kater et al. (37)	Total: 288 low-GC: 63 high-GC: 31	Venetoclax plus Rituximab	≥ 2nd line	low-GC: ≥ 3 genomic abnormalities high-GC: ≥ 5 genomic abnormalities	NA	Venetoclax plus Rituximab was superior in each CK category. GC had a major influence on clinical outcome.
		Bendamustine plus Rituximab				
Al-Sawaf et al. (38)	Total: 397 CK: 64	Venetoclax plus Obinutuzumab	1^st^ line	≥ 3 cytogenetic abnormalities	**2 year-PFS:** 78.9% **2-year-OS:** 88.2%	Venetoclax plus Obinutuzumab showed similar PFS and OS rates in patients with and without CK
		Chlorambucil plus Obinutuzumab			**2 year-PFS:** 36.6% **2-year-OS:** 82.7%	

CK, complex karyotype; GC, genomic complexity; OS, overall survival; PFS, progression free survival.

Ibrutinib-monotherapy, both in treatment-naïve and relapsed/refractory (R/R) CLL patients, demonstrated an overall response rate of approximately 90% (39). The presence of CK was associated with shorter duration of response [DOR, 31 months *vs*. not reached (NR)], PFS (31 months *vs*. NR), and median OS (54 months *vs*. NR) compared to patients with ≤2 cytogenetic aberrations (non-CK) (34, 39). That said, survival in R/R patients with CK appeared to be significantly influenced by the coexistence of del(17p) which was associated with decreased overall response rate (ORR, 82% *vs*. 100%; DOR, 23 *vs.* 53 months), PFS (25 *vs*. 55 months), and OS (32 months *vs*. NR).

Interestingly, a follow-up of the RESONATE study, a randomized comparison of ibrutinib to ofatumumab in previously treated CLL patients, demonstrated similar ORR (90% *vs*. 89%) and PFS (72% *vs*. 80%, log-rank p-value = 0.25) in ibrutinib patients with CK compared to those with non-CK (34). In striking contrast, patients enrolled on the ofatumumab arm carrying CK had a significantly lower ORR (6% *vs*. 33%, p <0.05) and PFS (0% *vs*. 10%) compared to the non-CK ones. The impact of ibrutinib on CLL with CK was further evaluated in treatment-naïve individuals within the Alliance A041202 study (35). Of note, the presence of baseline complexity did not portend a higher risk of progression or death in ibrutinib treated patients, raising questions whether baseline CK is biologically equivalent to CK due to clonal selection acquired after the administration of chemotherapy.

A randomized, double-blind, phase 3 study assessing the efficacy of idelalisib in combination with rituximab (IR) in patients with relapsed CLL and significant comorbidities, showed that IR provided ORR of 81 and 89% in CK and non-CK groups, respectively (odds ratio 0.5, p = 0.3509) (40). An extended study was designed to elucidate further the efficacy of IR in the presence of CK. Around 60% of patients with CK carried also TP53 aberrations compared to 43% of patients without CK. The median OS was prolonged in the CK-group treated with IR [median: 28.3 (range 16.6, NR) months), compared to patients with CK who received placebo/rituximab [median: 9.2 (range: 2.0, 53.5) months]. Of note, co-existence of CK and TP53 aberrations or del(11q) did not significantly affect survival in patients who received IR. That said, solid conclusion cannot be drawn since the sample size was small while the methodology for the detection of CK was not uniform (36).

Regarding venetoclax-based regimens, a 4-year clinical follow-up of MURANO study explored the predictive value of cytogenetic complexity in R/R CLL patients treated with venetoclax–rituximab (VenR) or bendamustine–rituximab (BR) (37). The authors followed the segregation in low-, intermediate- and high-CK, with the advent of MCA. Patients with non-CK demonstrated better PFS than those with either low-CK or high-CK status (HR, 2.0; 95% CI, 1.4 to 6.3; p = 0.025 and HR, 2.9; 95% CI, 1.1 to 3.6; p = 0.0057, respectively). Additionally, patients with high-CK showed a trend towards worse PFS *versus* those with fewer abnormalities (HR, 1.5; 95% CI, 0.7 to 3.4; p = 0.29).

he role of CK in venetoclax-based combination was also assessed in treatment-naïve patients following therapy with venetoclax–obinutuzumab (VenG) or cholambucil–obinutuzumab (ClbG) within the CLL14 trial (38). Not surprisingly, 32% of patients with CK carried also TP53 aberrations contrasting non-CK patients, (8%). U-CLL was present in similar proportions of patients with CK and non-CK. VenG was associated with significantly better responses independently of the presence of CK with ORRs of 82.4 and 87.3% (p= 0.42) for patients with CK and non-CK respectively. The complete eradication of the leukemic cells is a desired endpoint in CLL management usually translating in a better outcome (41–43). In the VenG arm, the rates of undetectable MRD (uMRD) were similar between patients with CK and non-CK in the peripheral blood (79.4% *vs* 77.1%; p = 1.0) and in the bone marrow (58.8% *vs*. 57.8%, p = 1.0). These high uMRD-rates were reflected into non-statistically significant differences between those groups in PFS (median, NR; 2-year-PFS rate, 78.9 and 91.1%, respectively; HR, 1.909; 95% CI, 0.806–4.520) and OS (median, NR; 2-year-OS rate, 88.2 and 93.2%; HR, 1.511; 95% CI, 0.496–4.600). Interestingly, no difference was neither observed within the CK cohort when the level of cytogenetic complexity was taken into consideration, with the number of aberrations (≥ or <5) not having any impact on clinical outcome. That said the number of patients with ≥5 aberrations was extremely low. Finally, TP53 aberrations did not have any impact on the outcome of patients with CK treated with VenG.

## Discussion

As the concept of precision medicine becomes part of the routine-management of CLL, the need for identifications of biomarkers, which may guide treatment choices, is imperative. These markers should be solid, reproducible and highly specific to the available treatment alternatives. Until today, TP53 aberrations and the somatic hypermutation status of the immunoglobulin heavy variable genes (IGHV) are the main disease-related features that shape the treatment algorithm in CLL. Several other genetic abnormalities have been associated with distinct clinical outcomes without however being incorporated in the clinical praxis. CK is a novel candidate biomarker that seems to be of significance regarding prognosis, but more importantly prediction, even in the era of the novel agents. However, several issues need to be further addressed before CK can be integrated in the clinical setting.

For many years, there were concerns regarding the applied methodology for the detection of CK. Today there is no doubt that with the advent of specific culture protocols, i.e., the addition of mitogens, CBA is a robust and reproducible methodology in CLL, and therefore, the obtained karyotypes are fully trustworthy and representative of the CLL clone. However, it is still unclear whether CMA can replace CBA and serve as a surrogate method for the detection of cytogenetic complexity. Indeed, CMA can be informative regarding the grade of complexity but adoption of definitions based on CBA, namely, low-CK *vs* high-CK should be followed with caution as the two methodologies have a number of differences that may affect their output.

When it comes to prognosis in cancer, it is highly common to apply prognostic indices, which are generated after the performance of statistical analysis taking into account a number of clinical and biological features. CLL is no exception (6). Over the years, numerous prognostic indices have been proposed. Nevertheless, their clinical utility has been questioned, since their actual applicability is limited (44, 45). CK has not thus far been assessed within the context of a prognostic index, mainly due to missing cytogenetic data for the great majority of the patients that have been included in such studies. Whether, the implementation of CK in the generation of prognostic indices has the potential to improve their applicability is still unknown.

Another issue that remains open is whether CK is indeed independent of other high-risk features mainly TP53 aberrations and U-CLL. Undoubtedly, patients with CK are enriched for TP53 aberrations and U-CLL with that enrichment reaching higher rates as the number of cytogenetic aberrations increases. That said, high-CK is even present in M-CLL as well as in patients without TP53 aberrations retaining its prognostic value. Therefore, the traditional claim that CK and TP53 aberrations always co-exist seems not to be true. Of note, in a meaningful proportion of CLL with CK, the presence of TP53 aberrations has been excluded even with the performance of highly sensitive methodologies that allow the detection of even small TP53 clones. One could therefore suggest that at least within a number of patients with CK, genomic instability could be independent of p53 biology. Coming to the interaction of CK and U-CLL, there are still many unaddressed issues. In general, U-CL is associated with high-risk genetic features with CK being no exception. However, CK has been reported even within M-CLL where it seems to be associated with worse clinical courses. This notion however does apply in all M-CLL since specific cytogenetic aberrations seem to overcome the negative impact of cytogenetic complexity. A typical example is M-CLL with CK carrying +12,+19, a subgroup accounting for 1-2% of all CLL that exhibits extremely indolent clinical behavior irrespectively of the grade of cytogenetic complexity (4).

In the context of clinical trials, the data regarding the impact of CK are still scarce, since the number of included cases is extremely low and therefore any extrapolation is quite uncertain. Therefore, more studies are needed in order to reach solid conclusions regarding the predictive value of CK. Nevertheless, the low number of high-CK (5%) at least at front-line treatment underscores the need for large population-studies, since it will be difficult to recruit the required number of patients with high-CK in the context of a clinical trial in order to reach statistical significance. Following this approach, it was recently reported that increasing cytogenetic complexity was an independent predictor of shorter PFS [HR1.07 (95% CI 1.04–1.10), p <0.0001] and overall survival [HR 1.09 (95% CI 1.05–1.12), p <0.0001] for patients treated with ibrutinib (46).

Taking into consideration the available data it is obvious that the heterogeneity of CLL extends even within the CK group with not all CKs being equivalent. The number of aberrations, the type of aberrations as well as the impact of clonal selection due to treatment are only few of the parameters that seem to impact on the clinical significance of CK in CLL. Therefore, it is highly urgent to obtain concrete guidelines for the interpretation of CBA and CMA findings in the clinical practice in order to reach consensus on the potential role of CK in the management of CLL. In conclusion, CK is a strong prognostic marker in CLL, while its predictive value remains unclear, especially in the era of novel agents.

## Author Contributions

All authors listed have made a substantial, direct, and intellectual contribution to the work and approved it for publication.

## Conflict of Interest

PB has received honoraria from Abbvie, Gilead, Janssen, and research grants from Gilead.

The remaining authors declare that the research was conducted in the absence of any commercial or financial relationships that could be construed as a potential conflict of interest.

## Publisher’s Note

All claims expressed in this article are solely those of the authors and do not necessarily represent those of their affiliated organizations, or those of the publisher, the editors and the reviewers. Any product that may be evaluated in this article, or claim that may be made by its manufacturer, is not guaranteed or endorsed by the publisher.
